# Are genes the missing link to detect and prognosticate RA-ILD?

**DOI:** 10.1093/rap/rkad023

**Published:** 2023-03-06

**Authors:** Marie Vermant, Tinne Goos, Stefan Gogaert, Diederik De Cock, Patrick Verschueren, Wim A Wuyts

**Affiliations:** Laboratory of Respiratory Diseases and Thoracic Surgery (BREATHE), Department of Chronic Diseases and Metabolism, KU Leuven, Leuven, Belgium; Pulmonology, University Hospitals Leuven, Leuven, Belgium; Laboratory of Respiratory Diseases and Thoracic Surgery (BREATHE), Department of Chronic Diseases and Metabolism, KU Leuven, Leuven, Belgium; Pulmonology, University Hospitals Leuven, Leuven, Belgium; Laboratory of Respiratory Diseases and Thoracic Surgery (BREATHE), Department of Chronic Diseases and Metabolism, KU Leuven, Leuven, Belgium; Pulmonology, University Hospitals Leuven, Leuven, Belgium; Biostatistics and Medical Informatics Research Group, Department of Public Health, Vrije Universiteit Brussel, Brussels, Belgium; Skeletal Biology and Engineering Research Center, Department of Development and Regeneration, KU Leuven, Leuven, Belgium; Rheumatology, University Hospitals Leuven, Leuven, Belgium; Laboratory of Respiratory Diseases and Thoracic Surgery (BREATHE), Department of Chronic Diseases and Metabolism, KU Leuven, Leuven, Belgium; Pulmonology, University Hospitals Leuven, Leuven, Belgium

**This editorial refers to ‘A genome-wide association study identifying single nucleotide polymorphisms in the *PPFIBP2* gene was predictive for interstitial lung disease in rheumatoid arthritis patients’ by Hayashi *et al.* [1].**

Approximately 10% of RA patients develop an interstitial lung disease (RA-ILD) during their disease course, although only a few studies have investigated the epidemiology in detail [2]. Patients with RA-ILD clinically present with dyspnoea and dry cough. They predominantly have a usual interstitial pneumonia pattern on CT [2]. The prognosis of RA-ILD with this CT pattern is comparable to idiopathic pulmonary fibrosis, resulting in a survival after ILD diagnosis of 3.2–10.2 years [2]. Current screening modalities and treatments for the respiratory complications of RA are insufficient even with the arrival of antifibrotic drugs for CTD-ILD [3]. Therefore, rapidly detecting RA-ILD is an unmet need.

Multiple clinical RA-ILD risk factors have been identified, including male sex, ever smokers, RF positivity and the presence of anti-citrullinated antibodies (Table 1) [2]. Furthermore, the following molecular biomarkers have already been linked with RA-ILD: epithelial cell-derived Krebs von den Lungen (KL-6) antigen, matrix metalloproteinase 7 (MMP-7), pulmonary and activation-regulated chemokine (PARC), surfactant protein D (SP-D), interferon-γ-inducible protein 10 (IP-10/CXCL10) and malondialdehyde–acetaldehyde adducts antibodies (anti-MAA) [4]. In addition, new prognostic genetic markers and risk stratification tools are being explored. Hayashi *et al*. [1] performed a genome-wide association study in search of new genetic biomarkers. They showed an association between the presence of the rs6578890 single nucleotide polymorphism (SNP) in the PPFIA binding protein 2 (*PPFIBP2*) gene and RA-ILD presence. rs6578890 had not previously been described. The authors propose two causal mechanisms. First, a role for the *PPFIBP2* gene, encoding liprin β-2, which plays a role in axon guidance and synapse development, is hypothesized. Second, a role for the distally located *CYB5R2* gene is suggested. The expression of this cytochrome member gene is influenced by the rs6578890 SNP. These pathways and their possible causal roles in RA-ILD pathophysiology must be further investigated.

**Table 1:** Overview of described RA-ILD risk factors in the literature

Clinical risk factors [2]		

Male		
Older age		
Ever smoker		

Serological biomarkers [4]		

RF		
Anti-CCP antibodies (anti-CCP)		
Anti-malondialdehyde-acetaldehyde antibodies (anti-MAA)		
Krebs von den Lungen-6 (KL6)		
C-X-C motif chemokine ligand 10 (IP-10/CXCL10)		
IL-18		
IL-13		
Surfactant protein D (SP-D)		
Lysyl oxidase-like 2 (LOXL2)		
Matrix metalloproteinase 7 (MMP-7)		
Pulmonary and activation-regulated chemokine (PARC)		

Genetic biomarkers [1, 5 ,6, 7, 10]		Described region

Genetic polymorphisms	*MUC5B* rs35705950	France, Greece, The Netherlands, USA, Japan, China
		Finland
	*RPA3/UMAS* rs12702634	Japan
	*PPFIBP2* rs6578890	Japan
Haplotypes	DRB1*16, DRB1*15, DQB1*06	Japan
Genetic mutations	TERT, RTEL1, PARN, SFTPC	France
Telomere length	<10th percentile	France

For all genetic risk factors, the region in which the risk factor was described was also included.

In addition to this possible new genetic marker, a shared genetic predisposition in RA-ILD and familial pulmonary fibrosis has previously been described [5]. In patients with RA-ILD, several mutations have been found in telomere-related and surfactant-related genes, similar to findings in patients with familial pulmonary fibrosis. Furthermore, short leucocyte telomere length (<10th percentile) has been associated with RA-ILD, progressive disease and early mortality [5]. Recently the rs12702634 variant in the *RPA3* (replication protein A3) and *UMAD1* (UBAP1-MVB12-associated domain containing 1) genes was reported to be associated with RA-ILD in a Japanese cohort [6]. These genes play an important role in the modulation of telomere elongation. Also, the functional MUC5B rs35705950 promoter variant has been identified as a risk factor for RA-ILD, whereas it was not associated with RA without ILD [7]. A large observational study showed a more than 10-fold elevated risk of ILD when having both the MUC5B promotor variant and RA when compared with the general population. Based on these results, it was suggested to use the MUC5B rs35705950 promotor variant for genomic risk stratification in patients with RA [8]. Surprisingly, in the recently published study by Hayashi *et al*. [1], no association was found with the MUC5B promotor variant rs35705950 and the presence of RA-ILD, but this could be due to the low penetrance of this variant in the Japanese population. As genetics and geography are closely linked, it is necessary to look at different predominant genomic biomarkers based on differences in geography [9].

All these biomarkers, both molecular and genetic, lack validation and have not yet been implemented in daily practice. An explanation for this can be found on multiple levels. Large, prospective patient cohorts are currently missing. Also, there is a scarcity of knowledge concerning RA-ILD pathogenesis and natural history, complicating the establishment of causal relationships and the differentiation of biomarkers for prognosis and development. Furthermore, these biomarkers need to be specific and cost-effective before implementation in practice. With the arrival of new antifibrotic therapeutic interventions, detecting RA-ILD early might become even more important, assuming these agents effectively decrease fibrotic disease progression. Currently there is no standardized screening method. Detection by high-resolution CT (HRCT) is performed on a case-by-case basis, where initial symptoms, such as a dry cough or dyspnoea upon exertion, can be masked by limited physical activity. Although the radiation dose in HRCT has been reduced over the years, there is still a small associated risk. Robust studies investigating other screening modalities are thus required, aiming to develop and subsequently implement a pragmatic, standardized screening protocol.

The study results by Hayashi *et al*. [1] must be interpreted with caution due to the relatively small sample size and geographical bias, yet also with enthusiasm, as this study sets the scene for further research. First, the association between RA-ILD and the *PPFIBP2* rs6578890 gene variant needs to be confirmed in larger study cohorts and different geographical regions. Second, the causal role of the *PPFIBP2* and *CYB5R2* genes in the development of ILDs needs to be explored. Third, a role for genomic biomarkers—both as a monogenetic test or as a polygenetic testing array—for detection of patients at risk for the development of RA-ILD and for further prognostic stratification based on the underlying pathogenic mechanism needs to be examined.

To conclude, a multimodal screening tool is needed, incorporating clinical, radiological, physiological, molecular and—as supported by this work—genetic factors. The tool should focus on the detection and, ideally, the prognosis of RA-ILD. The genetic panel will probably have to be adapted to geographical regions. Moreover, the risk stratification tool should be tested and validated in a large patient cohort and multiple geographical zones. Although lots of gaps in our knowledge regarding RA-ILD still need to be bridged, genes might constitute the possible missing link to reliably detect and prognosticate RA-ILD and could even provide a basis for personalized medicine.

## Data Availability

No new data were presented in this article.
